# Combination of Binary System with Ki67 Immunohistochemical Staining: a Reliable Grading Method for Oral Epithelial Dysplasia

**DOI:** 10.30476/DENTJODS.2021.91032.1546

**Published:** 2022-09

**Authors:** Negar Akafzadeh, Fereshteh Baghaei, Samira Derakhshan, Monir Moradzadeh Khiavi, Mohamadjavad Kharazifard, Pouyan Aminishakib

**Affiliations:** 1 Postgraduate Student of Pediatric Dentistry, Dept. of Pediatric Dentistry, School of Dentistry, Qazvin University of Medical Sciences, Qazvin, Iran; 2 Retired Professor, Dept. of Oral and Maxillofacial Pathology, School of Dentistry, Tehran University of Medical Sciences, Tehran, Iran; 3 Dept. of Oral and Maxillofacial Pathology, School of Dentistry, Tehran University of Medical Sciences, Tehran, Iran; 4 Researcher, Dental Research Center, Dentistry Research Institute, Tehran University of Medical Sciences, Tehran, Iran

**Keywords:** Ki-67 Antigen, Precancerous Conditions, Carcinoma in Situ

## Abstract

**Statement of the Problem:**

Oral epithelial dysplasia (OED) is a common potentially malignant lesion of oral cavity that should be managed to prevent likely malignant transformation.

**Purpose:**

Here, we present a combination of binary grading system with complementary immunohistochemical (IHC) staining for Ki67 biomarker to provide a reproducible OED grading system.

**Materials and Method:**

In this cross-sectional study, seventy out of one hundred OED specimens, which were accompanied by IHC stained microscopic slides for Ki67 antigen were evaluated by four independent oral pathologists. Both three-tier and binary grading systems based on WHO microscopic criteria were employed , blindly in a four-step method with at least two-week interval between each observation. Intra- and inter-observational reliability was assessed using Kappa statistical analysis.

**Results:**

OED diagnosis based on binary system showed significant intra-observer reliability comparing to three-tier system without biomarker. Moreover, OED diagnosis based on binary system using Ki67 biomarker showed significant inter-observer reliability comparing to diagnosis in three tier system and based on binary system without Ki67 biomarker showed significant inter-observer reliability comparing to diagnosis based on three-tier system without Ki67.

**Conclusion:**

Here, we found that application of IHC staining for Ki67 biomarker in binary system might provide a more reliable grading method for oral pathologist form different educational background.

## Introduction

Oral cancer is one of the most common cancers in human population with high morbidity and mortality rates [ 1 - 2 ]. Prediction of probability of malignant change in dysplastic oral epithelium, as a potentially malignant lesion is a clinical concern for both pathologists and clinicians, which has a great impact on therapeutic approaches [ 3 - 4 ]. Over the past decades, several grading systems of oral epithelial dysplasia (OED) have been introduced to provide a reliable classification method with sharply defined borders and significant reproducibility [ 6 - 7 ]. Although there is an agreement among investigators that all OEDs do not necessarily transform to an invasive carcinoma, it is inevitable to guide surgeons, facing this potentially malignant lesion, using a clearly interpretable scoring system, which is well-descriptive and fulfills the majority of microscopic features [ 8 - 9 ].

Most well-known grading systems are developed based on microscopic criteria, introduced by World Health Organization (WHO) in 2005 and revised in 2017 [ 10 ]. Considering these criteria, OED is scored in a three-graded system (mild, moderate and severe), according to the level of extension of microscopic criteria along epithelial tissue, or in a two-graded system (low-risk, high-risk) according to the severity of epithelial involvement by cellular atypia and architectural changes [ 11 ].

Despite all the efforts to propose a grading system with restricted histopathologic subjectivity, emerging of ancillary objective biomarkers may be necessary in routine laboratory practice for microscopic evaluation of the specimens, suspicious of epithelial dysplasia. Over recent years, a wide range of immunohistochemically (IHC) detectable proteins associated with risk of oral cancer initiation is proposed. These proteins are mostly depicted in cancer hallmarks [ 12 ]. Ki67, routinely used proliferation marker, is commonly utilized to make definite diagnosis in all types of cancers. Additionally, a recent study has shown that Ki-67 may be helpful in OED stratification by significant nuclear expression, particularly in high-risk dysplasia [ 13 ].

Among all OED grading systems and ancillary bio-markers, in the present investigation, we compared two, binary and three-tier WHO grading systems, with and without supplementary IHC study (proliferation marker) to assess their intra- and inter-observer reliabilities.

## Materials and Method

### Case collection

The present study was approved by Ethical Committee in Research of Tehran University of Medical Sciences (#IR.TUMS.DENTISTRY.REC.1396.4125).

The materials composed of 100 formalin-fixed paraffin-embedded specimens submitted in Oral and Maxillofacial Pathology Department, School of Dentistry, Tehran University of Medical Sciences (TUMS) during the period of ten years (1999 to 2019) with microscopic diagnoses of dysplasia, based on three-graded WHO scoring system (including 34 mild, 34 moderate and 32 severe dysplasia cases). All paraffin blocks and hematoxylin and eosin (H&E) stained microscopic slides were evaluated by one of the authors (N.A.) to approve the quantity and quality of the specimens.

### IHC staining process

The paraffin blocks with enough specimens (70 including 30 mild, 21 moderate and 19 severe dysplasia cases) were immunohistochemically stained for Ki67 biomarker (DAKO detection system, Denmark). Positive control consisted of appendix specimen and negative control was provided by omitting primary antibody during staining process.

### Examination process

**Figure 1:**
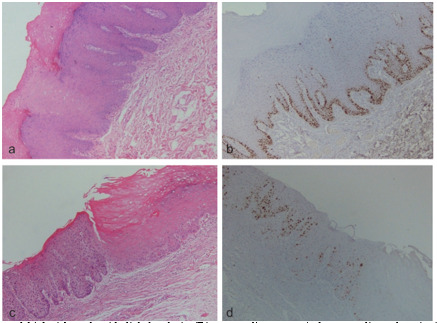
Low-risk and high-risk oral epithelial dysplasia (Binary grading system): hematoxylin and eosin (H&E) staining and immunohistochemical (IHC) staining for Ki67 biomarker (100× magnification). **a:** Low-risk oral epithelial dysplasia: H&E staining, **b:** IHC staining for Ki67, **c:** High-risk oral epithelial dysplasia: H&E staining, **d:** IHC staining for Ki67

All H&E (Figure 1a  and Figure 1c) and IHC (Figure 1b and Figure 1d) stained cases were coded by one of the authors (N.A.) before reviewing by the oral pathologists. Four independent oral pathologists from TUMS (P.A., F.B., S.D., M.M.K.) with different educational background, reviewed microscopic slides, separately, based on WHO criteria for epithelial dysplasia (Table 1) using light microscopy (Olympus CX31, Japan) as follows:

**Table 1:** World Health Organization (WHO) microscopic criteria for oral epithelial dysplasia

Architectural changes	Cellular changes
Irregular epithelial stratification	Abnormal variation in nuclear size (anisonucleosis)
Loss of polarity of basal cells	Abnormal variation in nuclear shape (nuclear pleomorphism)
Drop-shaped rete ridges	Abnormal variation in cell size (anisocytosis)
Increased number of mitotic figures	Abnormal variation in cell shape (cellular pleomorphism)
Abnormal superficial mitosis	Increased nuclear-cytoplasmic ratio
Premature keratinization in single cells (dyskeratosis)	Atypical mitotic figures
Keratin pearls within rete ridges	Increased number and size of nucleoli
Loss of epithelial cell cohesion	Hyperchromasia

• Step 1: All H&E stained microscopic slides (100 cases) were reviewed by oral pathologists based on both binary system and three-tier WHO classification (Table 2), blindly. The data was recorded for each system, separately.

**Table 2:** Three-tier vs. binary grading systems for oral epithelial dysplasia

Three-tier	Binary
**Mild:** Criteria for dysplasia limited to the lower third of the epithelium	**Low-risk:** less than four architectural changes or less than five cytological changes
**Moderate:** Criteria for dysplasia extending into the middle-third of the epithelium	**High-risk:** at least four architectural changes and five cytological changes
**Severe:** Criteria for dysplasia observed in greater than two-third of the epithelium	

• Step 2: Two weeks after step 1, all H&E stained microscopic slides (100 cases) were reviewed again by oral pathologists based on both binary system and three-tier WHO classification, blindly. The data was recorded again for each system, separately.

• Step 3: Four weeks after step 2, all H&E stained with accompanying IHC stained (for Ki67 biomarker) microscopic slides (70 cases) were reviewed by oral pathologists based on both binary system and three-tier WHO classification, blindly. The data was recorded for each system, separately.

• Step 4: Two weeks after step 3, all H&E stained with accompanying IHC stained (for Ki67 biomarker) microscopic slides (70 cases) were reviewed by oral pathologists based on both binary system and three-tier WHO classification, blindly. The data was recorded again for each system, separately.

### Statistical analysis

The collected data was analyzed using SPSS software 25. Intra- and inter-observer reliability was evaluated using weighted Kappa and statistic values less than 0.05 were considered significant. For multiple comparisons, Bonferroni adjustment was applied on p values [ 14 ].

## Results

### Intra-observer reliability

Statistical analysis of intra-observer reliability demonstrates the below findings (Table 3):

**Table 3:** Intra-observer reliability analysis of four independent observers (binary and three-tier grading systems with and without Ki67 IHC staining)

	Values	1^st^ observer	2^nd^ observer	3^rd^ observer	4^th^ observer
		1^st^ vs 2^nd^ observation	1^st^ vs 2^nd^ observation	1^st^ vs 2^nd^ observation	1^st^ vs 2^nd^ observation
Three-tier	K	0.238	0.317	0.345	0.367
	ASE	0.096	0.097	0.082	0.094
	*p* Value	0.004	0.00	0.000	0.000
Three-tier (with Ki67)	K	0.272	0.469	0.565	0.434
	ASE	0.098	0.095	0.093	0.095
	*p* Value	0.004	0.000	0.000	0.000
Binary	K	0.563	0.571	0.565	0.655
	ASE	0.099	0.095	0.107	0.094
	*p* Value	0.000	0.000	0.000	0.000
Binary (with Ki67)	K	0.792	0.657	0.757	0.691
	ASE	0.079	0.093	0.103	0.089
	*p* Value	0.000	0.000	0.000	0.000

K= Kappa Analysis, ASE: Asymptotic Standard Error

- Binary system in combination with Ki67 showed significant intra-observer reliability in comparison with three-tier system without Ki67.

- Binary system without Ki67 showed significant intra-observer reliability in comparison with three-tier system without Ki67.

- Twenty-five percent of the cases diagnosed using three-tier system in combination with Ki67 showed significant intra-observer reliability comparing to the cases diagnosed using three-tier system without Ki67.

Fifty percent of the cases diagnosed using binary system without Ki67, showed significant intra-observer reliability comparing to the cases diagnosed using three-tier system in combination with Ki67.

- Fifty percent of the cases diagnosed using binary system in combination with Ki67 showed significant intra-observer reliability comparing to the cases diagnosed using three-tier system in combination with Ki67.

Twenty-five percent of the cases diagnosed using binary system in combination with Ki67 showed significant intra-observer reliability comparing to the cases diagnosed using binary system without Ki67.

- OED diagnosis based on binary system (with or without Ki67 biomarker) showed significant intra-observer reliability comparing to three-tier system without biomarker.

### Inter-observer reliability

Statistical analysis of inter-observer reliability demonstrates the below findings (Table 4):

**Table 4:** Inter-observer reliability analysis of four independent observers (binary and three-tier grading systems with and without Ki67 IHC staining)

		Without Ki67			With Ki67		
		K	ASE	*p* Value	K	ASE	*p* Value
1^st^ vs 2^nd^ observer	Three-tier	0.381	0.093	0.000	0.381	0.094	0.000
1^st^ vs 3^rd^ observer		0.160	0.094	0.052	0.340	0.087	0.000
1^st^ vs 4^th^ observer		0.349	0.090	0.000	0.368	0.102	0.000
2^nd^ vs 3^rd^ observer		0.170	0.095	0.044	0.514	0.088	0.000
2^nd^ vs 4^th^ observer		0.344	0.091	0.000	0.350	0.098	0.000
3^rd^ vs 4^th^ observer		0.113	0.095	0.195	0.358	0.086	0.000
1^st^ vs 2^nd^ observer	Binary	0.514	0.102	0.000	0.509	0.110	0.000
1^st^ vs 3^rd^ observer		0.441	0.105	0.000	0.532	0.109	0.000
1^st^ vs 4^th^ observer		0.708	0.084	0.000	0.574	0.105	0.000
2^nd^ vs 3^rd^ observer		0.429	0.101	0.000	0.632	0.102	0.000
2^nd^ vs 4^th^ observer		0.514	0.099	0.000	0.403	0.117	0.001
3^rd^ vs 4^th^ observer		0.405	0.114	0.001	0.559	0.109	0.000

K= Kappa Analysis, ASE: Asymptotic Standard Error

- Two-third of the cases diagnosed using binary system (with/ without Ki67) showed significant inter-observer reliability comparing to the cases diagnosed using three-tier system without Ki67.

- Two-third of the cases diagnosed using binary system without Ki67 showed significant inter-observer reliability comparing to the cases diagnosed using three-tier system without Ki67.

- One-third of the cases diagnosed using three-tier system in combination with Ki67 showed significant inter-observer reliability comparing to the cases diagnosed using three-tier system without Ki67.

- No significant inter-observer reliability was found between three-tier system in combination with Ki67 comparing with diagnosis and binary system in combination with Ki67.

- No significant inter-observer reliability was found between binary system in combination with Ki67 comparing to binary system without Ki67.

- OED diagnosis based on binary system using Ki67 biomarker showed significant inter-observer reliability comparing to diagnosis in three tier system (with or without Ki67). Moreover, diagnosis based on binary system without Ki67 biomarker showed significant inter-observer reliability comparing to diagnosis based on three-tier system without Ki67 (Table not shown).

## Discussion

Oral squamous cell carcinoma commonly arises from OED, which necessitates therapeutic management of these potentially malignant lesions as a serious clinical challenge [ 15 ].

It seems that a universal, standardized grading system should be developed and utilized for OED classification with the following characteristics: First, it should be easily interpretable for clinicians/ surgeons to plan an effective treatment approach with minimum morbidity for the patients. Second, considering high incidence and prevalence of OED, it should be applicable in the vast majority of pathology laboratories, which do not have access to the advanced technologies to achieve molecular data. Third, it should restrict undesirable consequences of subjectivity in earlier introduced grading system; in fact, it should have both intra-observer and inter-observer reliabilities.

Here, we proposed a modified version of well-descried binary WHO grading system, in combination with a quite feasible IHC staining for Ki67 biomarker to reduce subjectivity and enhance reproducibility. 

A recently published study has confirmed that binary grading system is more reproducible and also more reliable prognostic scale comparing to widely used three-tier system [ 16 ]. Additionally, it seems clinicians can make definite decision to manage OED, especially more challenging cases with moderate dysplasia, with confidence using the binary system [ 9 ]. Our study showed similar results that excluding ancillary biomarker, binary system has both intra- and inter-observer reliability in comparison with three-tier system.

Although well-known WHO microscopic criteria for epithelial dysplasia are commonly used in most classifications, some studies have reported significant disagreement among observers. It is postulated that this inconsistency is not directly originated from the impairment of the microscopic criteria and is essentially based on the lack of rather uniform interpretation among observers. Therefore, the uniformity in interpretation is just likely among pathologists from the same institute [ 17 ], but it may not possible when consultant pathologist is from another laboratory with different experiences. Several biomarkers have been proposed to compensate limitations of binary grading system, including Ki67, CD105 and α-SMA [ 18 ], but it seems some of them cannot play a reliable complementary role for the scoring systems.

Ki67 protein demonstrates nuclear expression in all phases of cell cycle, excluding G0 phase. This protein is properly representative of cell proliferation activity and commonly used in determining behavior of the neoplasms to differentiate benign tumors from malignant ones [ 19 ]. Although some studies emphasize the importance of Ki67 expression level in oral epithelium, especially suprabasal level [ 13 ], we availed binary system of this biomarker by elucidating mitotic figures, nuclear size, nuclear shape, and increased nuclear-cytoplasmic ratio. We observed significant intra-observer reproducibility, in comparison with other three methods, when the observers have utilized binary system after reviewing both H&E and IHC stained microscopic slides. In addition, IHC stained sections for Ki67 biomarker clarify the distribution of mitotically active epithelial cells throughout whole thickness of the epithelium and consequently make the architecture of epithelium, uniformly interpretable for all observers from different centers with various experiences.

There are also several intervening factors with negative impact on making an accurate diagnosis in OED. The most important factor is inflammation in adjacent connective tissue, which induces cellular atypia mimicking true dysplastic changes [ 20 ]. In our study, all observers are recommended to consider inductive cellular atypia in cases with inflammation in superficial connective tissue.

It should additionally be mentioned that prognostic efficacy of the proposed grading method as an indicator of carcinomatous transformation is not evaluated in the present study which compel us for further investigation.

## Conclusion

In this study, we proposed a combination of binary grading system, based on WHO microscopic criteria, using both H&E and IHC stained for Ki67 protein microscopic slides. This method can simply improve objectivity of the binary system, which is well accepted by the majority of surgeons, and confine intra-/inter-observer discrepancies among pathologists from different educational background with unlike professional experiences.

## Acknowledgement

This study was D.D.S. thesis of Negar Akafzadeh and financially supported by School of Dentistry, Tehran University of Medical Sciences (Grant # 9211272032). 

We would like to thank Dr. Narges Hajiani (for her kind support to collect the formalin-fixed, paraffin-embedded specimens from laboratory archive) and Fatemeh Falahati Dowlatabadi (for her valuable support to provide high quality microscopic slides).

## Conflict of Interest

The authors declare that they have no conflicts of interests.
